# Aetiology of acute diarrhoea in children in Shanghai, 2015–2018

**DOI:** 10.1371/journal.pone.0249888

**Published:** 2021-04-08

**Authors:** Hailing Chang, Jiayin Guo, Zhongqiu Wei, Zheng Huang, Chuning Wang, Yue Qiu, Xuebin Xu, Mei Zeng

**Affiliations:** 1 Department of Infectious Diseases, Children’s Hospital of Fudan University, Shanghai, China; 2 Department of Microbiology, Changning District Center for Disease Control and Prevention, Shanghai, China; 3 Department of Microbiology, Shanghai Municipal Center for Disease Control and Prevention, Shanghai, China; Aga Khan University - Kenya, KENYA

## Abstract

Diarrhoea remains a major cause of childhood morbidity and mortality worldwide. This study aimed to monitor the aetiology of acute diarrhoea in children in Shanghai. Paediatric outpatients with acute diarrhoea were enrolled in the study from Jan 2015 to Dec 2018. Faecal samples were collected for testing. Enteric bacteria were identified and typed by culture and serotyping, respectively. Enteric viruses were identified by real-time PCR. Enteric pathogens were identified in 1572 (58.4%) of the 2692 enrolled children with acute diarrhoea. Viruses were detected more frequently than bacteria (41.3% versus 25.0%). Nontyphoidal *Salmonella* spp. (NTS) was the most common (10.3%) bacteria isolated, followed by enteropathogenic *Escherichia coli* (EPEC) (6.5%), enteroaggregative *Escherichia coli* (EAEC) (6.2%), *Campylobacter* spp. (3.6%), enterotoxigenic *Escherichia coli* (ETEC) (1.1%), *Shigella* spp. (0.2%), and enterohemorrhagic *Escherichia coli* (EHEC) (0.1%). Rotavirus was the most common (16.0%) virus detected, followed by norovirus (15.5%), adenovirus (7.2%), sapovirus (3.0%) and astrovirus (2.7%). Rotavirus, norovirus and NTS were the major pathogens responsible for diarrhoea in Shanghainese children. Improving uptake of the rotavirus vaccine and strengthening foodborne-pathogen prevention will aid in reducing the burden of diarrhoeal disease in children in Shanghai.

## Introduction

Diarrhoea is a major health problem in children and responsible for approximately 8.6% of paediatric deaths worldwide [1–3]. Due to continuous improvements in sanitation, hygiene and water supply safety, the diarrhoea-related death rate in children aged less than 5 years in China has decreased remarkably from 4.9% in 2000 to 3.2% in 2015 [4, 5]. China is a developing country with unbalanced regional economic development and medical care systems. Developing areas often have inadequate sanitation facilities, a lack of awareness of personal hygiene and a shortage of well-trained health workers, which puts children in developing areas at greater risk of diarrhoea than those in developed areas [6]. Previous studies have revealed that viral diarrhoea was more common than bacterial diarrhoea in developed areas compared with developing areas in China [7]. However, continuous monitoring data for multiple enteric pathogens are insufficient based on standardized microbiological laboratory detection methods.

The aetiology and epidemiology of infectious diarrhoea vary by time and location. We conducted a surveillance study over 4 years in paediatric outpatients with acute diarrhoea in Shanghai to inform policies and interventions targeted at preventing childhood diarrhoea.

## Materials and methods

### Case definition and enrollment

This surveillance study was conducted from Jan 2015 to Dec 2018 in the outpatient clinic at the Children’s Hospital of Fudan University, the largest tertiary paediatric teaching hospital in Shanghai. Diarrhoea was defined as at least three abnormally loose stools in the previous 24 hours, and an episode of diarrhoea is defined as diarrhoea onset beginning after at least 7 diarrhoea-free days and ending when diarrhoea is not present for 7 days [8]. Acute diarrhoea was defined as episodes of diarrhoea lasting for less than 14 days. The inclusion criteria for case enrolment were as follows: (1) children aged <18 years old; (2) children with diarrhoeal symptom onset 7 days before the hospital visit, which maximized the opportunity to identify the responsible pathogen; (3) children who resided in Shanghai; (4) children with adequate fresh stool specimens (at least l mL), which were collected for bacterial inoculation and virus detection; and (5) children who did not take antibiotics before enrolment. Children were excluded from the study if they had a known history of inflammatory bowel disease, food allergy, malnutrition, or other accompanying acute diseases.

### Sample collection and laboratory methods

Paediatric outpatients with a complaint of acute diarrhoea were enrolled every Monday and Wednesday, and the first 5–10 patients meeting the inclusion criteria were enrolled per sampling day. Verbal consent was obtained by the principal investigators through a face-to-face conversation with children’s legal guardian during sample collection. Clinical specimens and data were approved for use in this study by the Research Ethics Committee of the Children’s Hospital of Fudan University [(2014) No. 019].

Fresh faecal samples were collected and immediately preserved in Cary-Blair transport media (Shanghai Comagal Microbial Technology Co., Shanghai, China). The remaining portion of the stool sample was kept in Dulbecco’s modified Eagle’s medium at -4°C for virus detection. The samples were transported to the reference microbiological laboratory at the Changning District Center for Disease Control and Prevention within 24 hours for pathogen detection.

The enteropathogens tested in this study include five phenotypes of diarrhoeagenic *Escherichia coli* (DEC), nontyphoidal *Salmonella* spp. (NTS), *Shigella* spp., *Campylobacter* spp., *Yersinia enterocolitica*, pathogenic *Vibrio* spp., *Aeromonas* spp., rotavirus, norovirus Group I (G I) and Group II (G II), adenovirus, sapovirus and astrovirus. We did not include parasites and *Clostridium difficile* in the test panel because these two pathogens are not common pathogens of diarrhoea in Shanghainese children. Bacterial pathogens were identified as described in previous studies [9, 10]. Viruses were identified based on multiplex quantitative real-time PCR using BioPerfectus kits (Jiangsu BioPerfectus Technologies Co., Ltd., Jiangsu, China). All procedures were strictly followed according to the manufacturer’s instructions. Coinfection was defined as two or more different pathogens or different subtypes detected in one patient.

### Statistical analysis

Data were analysed using Stata software (Version 12.0; Stata Corp, College Station, TX). Odds ratios (ORs) and 95% confidence intervals (CIs) were calculated using logistic regression analyses controlled for sex to evaluate differences in the prevalence rates of specific enteropathogens by age group (age ≥60 months was used as the reference group).

## Results

A total of 2692 children with acute diarrhoea were enrolled in the study. The mean age of the enrolled children was 21.7 months (median age, 12.7 months, range: 0.3 months- 204 months), and 61.2% of the patients were male. The majority of enrolled children were younger than 3 years, accounting for 84.5% (Table 1).

**Table 1:** Demographic characteristics of enrolled outpatients with acute diarrhoea in Shanghai from 2015 to 2018 [n (%)].

	2015 (n = 790)	2016 (n = 605)	2017 (n = 633)	2018 (n = 664)	Total (n = 2692)
Male	481(60.9)	369 (61.0)	388 (61.3)	410 (61.7)	1648 (61.2)
Female	309 (39.1)	236 (39.0)	245 (38.7)	254 (38.3)	1044 (38.8)
Age (months)					
0–5	131 (16.6)	102 (16.9)	87 (13.7)	72 (10.8)	392 (14.6)
6–11	265 (33.5)	192 (31.7)	205 (32.4)	173 (26.1)	835 (31.0)
12–35	287 (36.3)	224 (37.0)	246 (38.9)	289 (43.5)	1046 (38.9)
36–59	54 (6.8)	45 (7.4)	47 (7.4)	57 (8.6)	203 (7.5)
≥60	53 (6.7)	42 (6.9)	48 (7.6)	73 (11.0)	216 (8.0)

Overall, enteropathogens were detected in 1572 (58.4%) of the 2692 enrolled cases. Rotavirus and norovirus were the most frequently identified viruses with a prevalence of 16.0% and 15.5%, respectively. Ninety-seven percent of norovirus belonged to GII. Adenovirus was relatively common with a prevalence of 7.2%. The prevalence of rotavirus and norovirus showed declining trends, while the prevalence of adenovirus showed an increasing trend from 2015 to 2018. Sapovirus and astrovirus caused sporadic gastroenteritis in children (Table 2).

**Table 2:** The distribution of enteric pathogens in Shanghainese children with acute diarrhoea from 2015 to 2018 [n (%)].

	2015 (n = 790)	2016 (n = 605)	2017 (n = 633)	2018(n = 664)	Total (n = 2692)
Rotavirus	142 (18.0)	105 (17.4)	107 (16.9)	77 (11.6)	431 (16.0)
Norovirus	137 (17.3)	103 (17.0)	98 (15.5)	80 (12.0)	418 (15.5)
GI	5 (0.6)	3 (0.5)	1 (0.2)	3 (0.4)	12 (0.4)
GII	132 (16.7)	100 (16.5)	97 (15.3)	77 (11.6)	406 (15.1)
Adenovirus	47 (5.9)	33 (5.5)	51 (8.1)	62 (9.3)	193 (7.2)
Sapovirus	31 (3.9)	13 (2.1)	17 (2.7)	19 (2.9)	80 (3.0)
Astrovirus	12 (1.5)	8 (1.3)	26 (4.1)	27 (4.1)	73 (2.7)
Nontyphoidal *Salmonella*	67 (8.5)	63 (10.4)	62 (9.8)	85 (12.8)	277 (10.3)
EAEC	38 (4.8)	38 (6.3)	52 (8.2)	40 (6.0)	168 (6.2)
EPEC	39 (4.9)	37 (6.1)	37 (5.8)	63 (9.5)	176 (6.5)
ETEC	8 (1.0)	9 (1.5)	6 (0.9)	7 (1.1)	30 (1.1)
EHEC	0 (0)	0 (0)	2 (0.3)	1 (0.1)	3 (0.1)
*Campylobacter*	44 (5.6)	15 (2.5)	15 (2.4)	23 (3.5)	97 (3.6)
*Shigella*	4 (0.5)	1 (0.2)	0 (0)	0 (0)	5 (0.2)
Co-infection	99 (12.5)	77 (12.7)	85 (13.4)	89 (13.4)	350 (13.0)

NTS was the most common bacteria, with a prevalence of 10.3%, followed by enteropathogenic *Escherichia coli* (EPEC) (6.5%), enteroaggregative *Escherichia coli* (EAEC) (6.2%), *Campylobacter* spp. (3.6%), enterotoxigenic *Escherichia coli* (ETEC) (1.1%), *Shigella* spp. (0.2%), and enterohemorrhagic *Escherichia coli* (EHEC) (0.1%). NTS isolates included 37 serovars, with *S*. Typhimurium and Enteritidis accounting for 39.0% and 25.3%, respectively, C*ampylobacter* isolates included *C*. *jejuni* (96.9%) and *C*. *coli (3*.*1%)*, and *Shigella* isolates were all *S*. *sonnei*. None of three EHEC strains were O157:H7 or O104:H4. No *Yersinia enterocolitica*, *Aeromonas* spp., *Vibrio* spp. or enteroinvasive *Escherichia coli* (EIEC) isolates were identified.

Compared with children aged ≥60 months, children aged 6–59 months had a higher risk of rotavirus infection. Norovirus was less commonly detected in children <6 months (OR: 0.2, 95% CI: 0.1–0.4). Children aged 12–35 months had an increased risk of DEC infection (OR: 1.6, 95% CI: 1.0–2.6). Children aged <6 months were less likely to have DEC infection (OR: 0.5, 95% CI: 0.3–0.9) and NTS (OR: 0.4, 95% CI: 0.2–0.7) than those aged > 6 months. Children <36 months were less likely to have *Campylobacter* infection than those aged > 36 months (Table 3).

**Table 3:** Age-specific prevalence of enteropathogens in children with acute diarrhoea.

Pathogen	0–5 months (n = 392)		6–11 months (n = 835)		12–35 months (n = 1046)		36–59 months (n = 203)		≥60 months^#^ (n = 216)	
	n (%)	OR (95% CI)	n (%)	OR (95% CI)	n (%)	OR (95% CI)	n (%)	OR (95% CI)	n (%)	OR
Rotavirus	38 (9.7)	1.4 (0.8–2.7)	130 (15.6)	2.5 (1.4–4.3)	217 (20.7)	3.5 (2.0–6.0)	31 (15.3)	2.4 (1.3–4.6)	15 (6.9)	1
Norovirus	17 (4.3)	0.2 (0.1–0.4)	134 (16.0)	1.0 (0.7–1.5)	202 (19.3)	1.3 (0.8–1.9)	30 (14.8)	0.9 (0.5–1.5)	35 (16.2)	1
Adenovirus	14 (3.6)	0.5 (0.2–1.2)	48 (5.7)	0.9 (0.5–1.7)	97 (9.3)	1.5 (0.8–2.7)	22 (10.8)	1.7 (0.8–3.7)	12 (5.6)	1
Sapovirus	3 (0.8)	0.3 (0.1–1.1)	14 (0.17)	0.6 (0.2–1.6)	49 (4.7)	1.7 (0.7–4.1)	8 (3.9)	1.4 (0.5–4.2)	6 (2.8)	1
Astrovirus	7 (1.8)	0.7 (0.3–1.9)	17 (2.0)	0.9 (0.4–2.1)	37 (3.5)	1.5 (0.7–3.4)	6 (3.0)	1.4 (0.5–3.8)	7 (3.2)	1
DEC	23 (5.9)	0.5 (0.3–0.9)	90 (10.8)	1.0 (0.6–1.6)	177 (16.9)	1.6 (1.0–2.6)	28 (13.8)	1.3 (0.7–2.3)	24 (11.1)	1
NTS	13 (3.3)	0.4 (0.2–0.7)	71 (8.5)	1 (0.6–1.7)	139 (13.3)	1.6 (0.9–2.6)	34 (16.7)	2.0 (1.1–3.8)	20 (9.3)	1
*Shigella*	0 (0)	NA	0 (0)	NA	2 (0.2)	NA	1 (0.5)	NA	2 (0.9)	NA
*Campylobacter*	3 (0.8)	0.1 (0.02–0.3)	10 (1.2)	0.1 (0.1–0.3)	48 (4.6)	0.5 (0.3–0.9)	18 (8.9)	1.1 (0.5–2.1)	18 (8.3)	1

# Children ≥60 months of age was the reference group.

NA indicates not applicable

**Fig 1:**
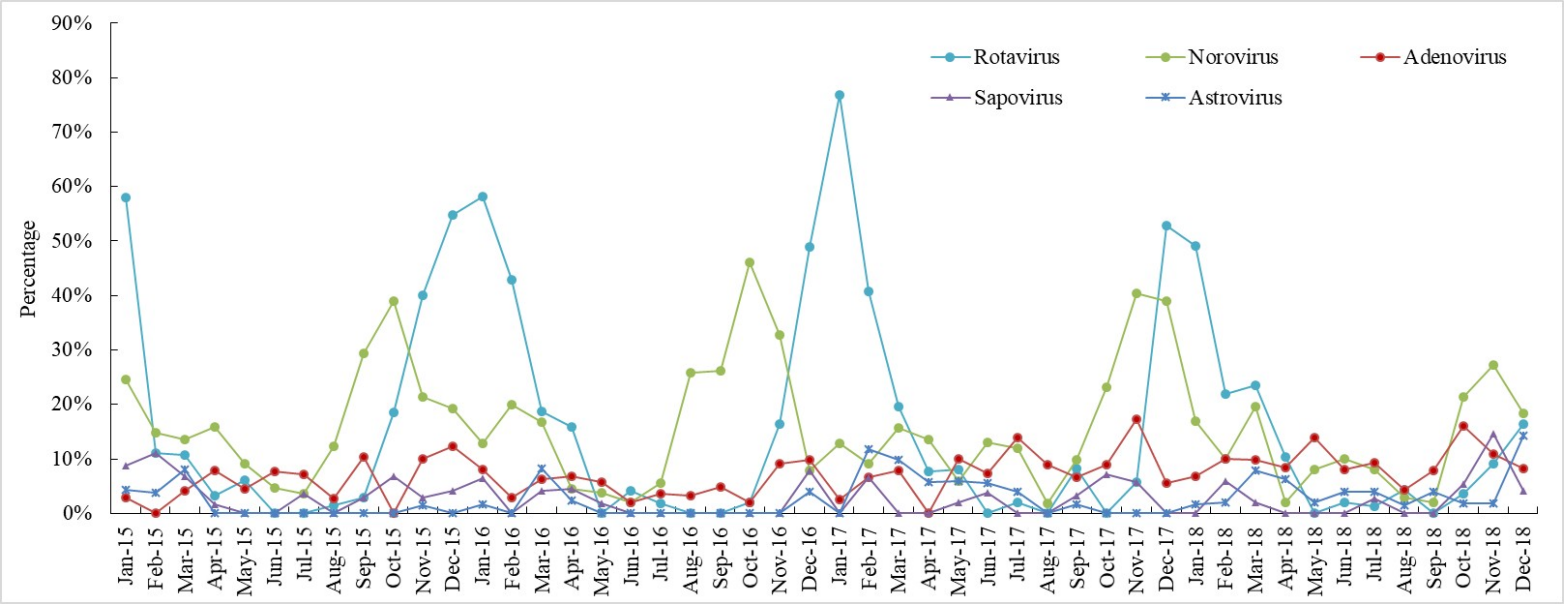
Seasonal prevalence of viruses in children with acute diarrhoea in Shanghai.

**Fig 2:**
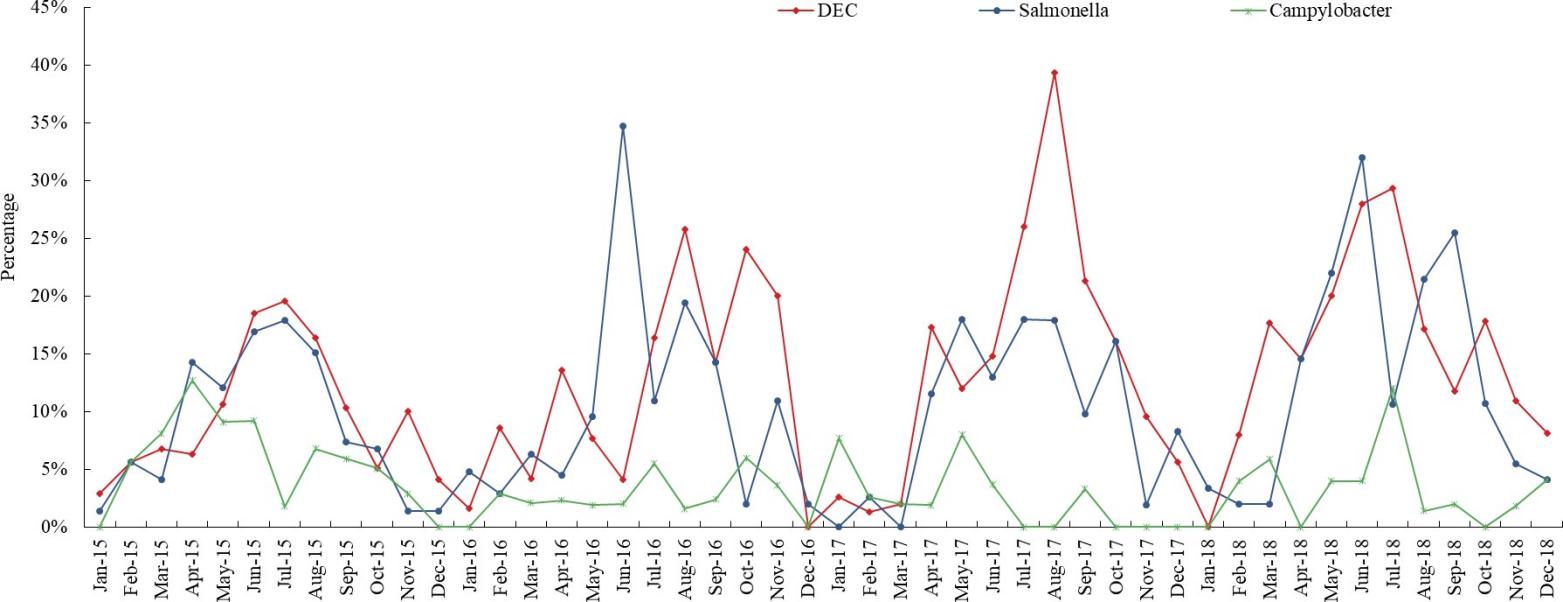
Seasonal prevalence of bacteria in children with acute diarrhoea in Shanghai.

The prevalence of enteric viruses usually peaked in autumn (September to November) and winter (December to February), but the seasonality of adenoviruses was not apparent (Fig 1). Norovirus infection usually peaked from August to November, prior to the rotavirus infection peak from November to February. The prevalence of enteric bacteria usually peaked in summer (June to August) and autumn (Fig 2).

## Discussion

In this surveillance study, enteropathogens were detected in 58.4% of children with acute diarrhoea in Shanghai; this result was significantly higher than the national average (44.6%) based on pooled data from 92 surveillance network laboratories [11]. The variation in the prevalence of enteropathogens could be related to laboratory detection capacity and techniques in our country. In addition, the epidemiological profiles of aetiological agents usually differ among different areas. For instance, 75.2% of diarrhoeal episodes in paediatric outpatients in Vietnam were attributable to infectious diarrhoea, with higher prevalence rates of rotavirus (41.6%) and norovirus (17.6%) infections [12]. The overall prevalence of enteropathogens in this study was consistent with that in a study in Salt Lake City (52%) [13]. However, the prevalence rates of rotavirus (3.7%) and NTS (1.9%) were very low, and the prevalence of *Clostridium difficile* (13.6%) was higher in Salt Lake City than in Shanghai [13]. Our findings showed that rotavirus, norovirus and NTS were the major aetiological agents responsible for acute diarrhoea annually in Shanghainese children.

The World Health Organization recommends prioritizing vaccination against rotavirus-related diarrhoea in 2008 based on the global disease burden [14]. The disease burden imposed by rotavirus-related diarrhoea has decreased remarkably in countries in which rotavirus vaccination has been included in the national immunization programme [15]. Although the Lanzhou lamb-derived rotavirus vaccine has been used in China since 2001, rotavirus remains the leading cause of diarrhoea in Chinese children [9, 11, 16]. The pentavalent rotavirus vaccine was introduced in China in September 2018, but the coverage among young infants was very low (personal communication with the CDC) in 2018. It is important to monitor the changing trends of rotavirus-related diarrhoea and diarrhoeal diseases as the demand and uptake of new rotavirus vaccines are increasing among infants in China.

The prevalence of norovirus infection was similar to that of rotavirus infection in this study. However, the peak of norovirus-related diarrhoea usually appeared earlier than that of rotavirus-related diarrhoea in Shanghai. Norovirus infection has replaced rotavirus infection as the leading cause of paediatric gastroenteritis requiring medical attention in countries with successful implementation of rotavirus vaccination, such as the US and Nicaragua [17–19]. Human noroviruses have three genogroups (GI, GII and GIV)) and numerous genotypes within each genogroup. It has been demonstrated that genogroup II, genotype 4 (GII.4), can cause epidemics and sometimes global pandemics of acute gastroenteritis when a new variant emerges after marked antigenic drift [20]. Thus, monitoring changes in the trend of norovirus activity is important to predict the potential emergence of new GII.4 variants and other recombinants. Considering the public health impacts of norovirus gastroenteritis outbreaks in communities, candidate norovirus vaccines are under development [21]. Adenovirus, sapovirus and astrovirus infections accounted for 12.9% of diarrhoeal symptoms in paediatric outpatients. Although the roles of these three viruses in community outbreaks are minor, they should not be neglected because these viruses are sometimes associated with outbreaks in semiclosed communities in all age groups [22, 23]. We observed that the prevalence rates of adenovirus and astrovirus increased in 2017 and 2018 compared to 2015 and 2016. Whether the shift in the prevalent serotypes was associated with increased adenovirus and astrovirus activities should be further analysed [21, 24, 25]; additional virus serotyping could help us understand this shifting pattern.

In this study, NTS was the third most common pathogen, accounting for 10.3% of diarrhoea cases, and was the leading bacterial pathogen in paediatric outpatients with diarrhoea. Globally, NTS is an important pathogen responsible for sporadic gastroenteritis and foodborne gastroenteritis outbreaks, and children <5 years of age are most susceptible, especially in developed countries [26, 27]. The annual prevalence of NTS from 2015–2018 was stable, with *S*. Typhimurium and *S*. Enteritidis predominating. Previous studies have suggested that NTS is spread through contaminated food, including eggs, pork, chicken, beef and vegetables [28]. Currently, the priority strategy for the control of NTS-related diarrhoea is strengthening food safety, which has reduced the incidence of NTS-related foodborne diseases in the US and some European countries [27, 28]. Although China had established measures to strengthen food and environmental monitoring and management, food contamination at certain steps in the supply chain, wet markets and supermarkets can result in NTS transmission and infection in humans. *Campylobacter* infection is a leading cause of foodborne illness in the US, and the incidence rate of *Campylobacter* infection in children aged 0–4 years was more than double the overall incidence rate [29]. In Shanghai, the prevalence of *Campylobacter* infection in children was relatively low, and the prevalence of shigellosis in children was extremely low. Urbanization and improvements in public sanitation have resulted in remarkable decreases in campylobacteriosis and shigellosis in China, which were the most common bacterial pathogens responsible for diarrhoea in Chinese children in the 1990s [30]. *Campylobacter* infection is usually linked with exposure to chickens and their products [31]. In 2013, the Chinese government started strengthening the management of live poultry markets to prevent the transmission of avian influenza, potentially reducing the transmission of *Campylobacter* from chickens to humans.

DEC was detected in 12.7% of Shanghainese children with acute diarrhoea, and most DEC isolates were EAEC and EPEC, consistent with studies conducted in Israel [32]. However, our previous case-control study conducted in 2014 did not demonstrate the pathogenic significance of EAEC and EPEC [9]. Therefore, we are uncertain about the roles of EAEC and EPEC as aetiological agents of infectious diarrhoea in Shanghainese children. Previous studies have shown that ETEC is the leading cause of travellers’ diarrhoea and a major cause of diarrhoeal disease, especially among children, in lower-income countries [33]. Interestingly, ETEC was not a common pathogen responsible for diarrhoea in Shanghainese children, even though it is a common cause of diarrhoea in adults in Shanghai [10]. This may be because a safe water supply and good public sanitation largely reduce exposure to ETEC in children in Shanghai, and young children are less likely to travel and eat outside. In addition, EHEC was rarely detected in Shanghainese children, and highly virulent O157:H7 and O104:H4 strains were not detected. EHEC is mainly transmitted through exposure to contaminated beef, and the O157:H7 and O104:H4 strains are known to cause outbreaks and severe diseases [34]. Usually, beef and medium-cooked meat are not frequently consumed by young Chinese children; thus, the chance of acquiring EHEC infection is very low.

## Conclusions

Infectious diarrhoea remains a major cause of diarrhoeal illnesses in Shanghainese children. The disease burden of diarrhoeal illnesses will decrease with increased coverage of rotavirus vaccination in susceptible infants and strengthened interventions targeting foodborne illnesses in Shanghai and other areas of China. The findings of this study may aid public health policy-makers in formulating effective strategies for the control and prevention childhood diarrhoea in China.

## References

[1] Fischer Walker CL, Perin J, Aryee MJ, Boschi-Pinto C, Black RE. Diarrhea incidence in low- and middle-income countries in 1990 and 2010: a systematic review. BMC Public Health 2012;12:220. 10.1186/1471-2458-12-220 22436130 PMC3323412

[2] Reiner RC Jr, Graetz N, Casey DC, Troeger C, Garcia GM, Mosser JF, et al. Variation in Childhood Diarrheal Morbidity and Mortality in Africa, 2000–2015. N Engl J Med 2018;379:1128–1138. 10.1056/NEJMoa1716766 30231224 PMC6078160

[3] Liu L, Oza S, Hogan D, Chu Y, Perin J, Zhu J, et al. Global, regional, and national causes of under-5 mortality in 2000–15: an updated systematic analysis with implications for the Sustainable Development Goals. Lancet 2016;388:3027–3035. 10.1016/S0140-6736(16)31593-8 27839855 PMC5161777

[4] Song P, Theodoratou E, Li X, Liu L, Chu Y, Black RE, et al. Causes of death in children younger than five years in China in 2015: an updated analysis. J Glob Health 2016;6:020802. 10.7189/jogh.06.020802 28028436 PMC5140075

[5] Wang Y, Miao L, Qian Y, Liang J, Wu Y, Zhu J, et al. Analysis of under 5 years old children mortality and the leading death cause in China from 1996 to 2000. Chin J Prev Med 2005;39:260–264. 16194383

[6] Thapar N, Sanderson IR. Diarrhoea in children: an interface between developing and developed countries. Lancet 2004;363:641–653. 10.1016/S0140-6736(04)15599-2 14987892

[7] Wang X, Wang J, Sun H, Xia S, Duan R, Liang J, et al. Etiology of Childhood Infectious Diarrhea in a Developed Region of China: Compared to Childhood Diarrhea in a Developing Region and Adult Diarrhea in a Developed Region. PLoS One 2015;10:e0142136. 10.1371/journal.pone.0142136 26528820 PMC4631449

[8] Kotloff KL, Nataro JP, Blackwelder WC, Nasrin D, Farag TH, Panchalingam S, et al. Burden and aetiology of diarrhoeal disease in infants and young children in developing countries (the Global Enteric Multicenter Study, GEMS): a prospective, case-control study. Lancet 2013;382:209–222. 10.1016/S0140-6736(13)60844-2 23680352

[9] Chang H, Zhang L, Ge Y, Cai J, Wang X, Huang Z, et al. A Hospital-based Case-control Study of Diarrhea in Children in Shanghai. Pediatr Infect Dis J 2017;36:1057–1063. 10.1097/INF.0000000000001562 28178108

[10] Huang Z, Xu H, Guo J, Huang X, Li Y, Hou Q, et al. Assessment and application of a molecular diagnostic method on the detection of four types of diarrheagenic Escherichia coli. Chin J Epidemiol 2013;34:614–617 24125616

[11] Yu J, Jing H, Lai S, Xu W, Li M, Wu J, et al. Etiology of diarrhea among children under the age five in China: Results from a five-year surveillance. The Journal of infection 2015;71:19–27. 10.1016/j.jinf.2015.03.001 25753104 PMC4667737

[12] Thompson CN, Phan MV, Hoang NV, Minh PV, Vinh NT, Thuy CT, et al. A prospective multi-center observational study of children hospitalized with diarrhea in Ho Chi Minh City, Vietnam. Am J Trop Med Hyg 2015;92:1045–1052. 10.4269/ajtmh.14-0655 25802437 PMC4426562

[13] Stockmann C, Pavia AT, Graham B, Vaughn M, Crisp R, Poritz MA, et al. Detection of 23 Gastrointestinal Pathogens Among Children Who Present With Diarrhea. J Pediatric Infect Dis Soc 2017;6:231–238. 10.1093/jpids/piw020 27147712 PMC5907859

[14] World Health Organization. Meeting of the immunization Strategic Advisory Group of Experts, November 2007—conclusions and Recommendations. Wkly Epidemiol Rec 2008;83:1–16. 18175408

[15] Troeger C, Khalil IA, Rao PC, Cao S, Blacker BF, Ahmed T, et al. Rotavirus Vaccination and the Global Burden of Rotavirus Diarrhea Among Children Younger Than 5 Years. JAMA Pediatr 2018;172:958–965. 10.1001/jamapediatrics.2018.1960 30105384 PMC6233802

[16] Liu N, Xu Z, Li D, Zhang Q, Wang H, Duan ZJ. Update on the disease burden and circulating strains of rotavirus in China: a systematic review and meta-analysis. Vaccine 2014;32:4369–4375. 10.1016/j.vaccine.2014.06.018 24958704

[17] Riddle MS, Chen WH, Kirkwood CD, MacLennan CA. Update on vaccines for enteric pathogens. Clin Microbiol Infect 2018;24:1039–1045. 10.1016/j.cmi.2018.06.023 29964231

[18] Payne DC, Vinjé J, Szilagyi PG, Edwards KM, Staat MA, Weinberg GA, et al. Norovirus and medically attended gastroenteritis in U.S. children. N Engl J Med 2013;368:1121–1130. 10.1056/NEJMsa1206589 23514289 PMC4618551

[19] Bucardo F, Reyes Y, Svensson L, Nordgren J. Predominance of norovirus and sapovirus in Nicaragua after implementation of universal rotavirus vaccination. PLoS One 2014;9:e98201. 10.1371/journal.pone.0098201 24849288 PMC4029982

[20] Bányai K, Estes MK, Martella V, Parashar UD. Viral gastroenteritis. Lancet 2018;392:175–186. 10.1016/S0140-6736(18)31128-0 30025810 PMC8883799

[21] Cortes-Penfield NW, Ramani S, Estes MK, Atmar RL. Prospects and Challenges in the Development of a Norovirus Vaccine. Clin Ther 2017;39:1537–1549. 10.1016/j.clinthera.2017.07.002 28756066 PMC5776706

[22] Wang J, Li Y, Kong X, Li H, Zhang Q, Jin M, et al. Two gastroenteritis outbreaks caused by sapovirus in Shenzhen, China. J Med Virol 2018;90:1695–1702. 10.1002/jmv.25236 29882310

[23] Tan Y, He WT, Chen MM, Mo JJ, Ju Y, Chen M. An outbreak of human astrovirus lineage 1b in a middle school in Guangxi, Southern China in 2017. Chin Med J (Engl) 2019;132:336–338. 30681500 10.1097/CM9.0000000000000072 PMC6595803

[24] Bosch A, Pintó RM, Guix S. Human astroviruses. Clin Microbiol Rev 2014;27:1048–1074. 10.1128/CMR.00013-14 25278582 PMC4187635

[25] Lion T. Adenovirus infections in immunocompetent and immunocompromised patients. Clin Microbiol Rev 2014;27:441–462. 10.1128/CMR.00116-13 24982316 PMC4135893

[26] Majowicz SE, Musto J, Scallan E, Angulo FJ, Kirk M, O’Brien SJ, et al. The global burden of nontyphoidal Salmonella gastroenteritis. Clin Infect Dis 2010;50:882–889. 10.1086/650733 20158401

[27] Tack DM, Marder EP, Griffin PM, Cieslak PR, Dunn J, Hurd S, et al. Preliminary Incidence and Trends of Infections with Pathogens Transmitted Commonly Through Food—Foodborne Diseases Active Surveillance Network, 10 U.S. Sites, 2015–2018. MMWR Morb Mortal Wkly Rep 2019;68:369–373. 10.15585/mmwr.mm6816a2 31022166 PMC6483286

[28] Pires SM, Vieira AR, Hald T, Cole D. Source attribution of human salmonellosis: an overview of methods and estimates. Foodborne Pathog Dis 2014; 11:667–676. 10.1089/fpd.2014.1744 24885917 PMC10938214

[29] Geissler AL, Bustos Carrillo F, Swanson K, Patrick ME, Fullerton KE, Bennett C, et al. Increasing Campylobacter Infections, Outbreaks, and Antimicrobial Resistance in the United States, 2004–2012. Clin Infect Dis 2017;65:1624–1631. 10.1093/cid/cix624 29020144

[30] Huilan S, Zhen LG, Mathan MM, Mathew MM, Olarte J, Espejo R, et al. Etiology of acute diarrhoea among children in developing countries: a multicentre study in five countries. Bull World Health Organ 1991;69:549–555. 1659953 PMC2393250

[31] Silva J, Leite D, Fernandes M, Mena C, Gibbs PA, Teixeira P. Campylobacter spp. as a Foodborne Pathogen: A Review. Front Microbiol 2011;2:200. 10.3389/fmicb.2011.00200 21991264 PMC3180643

[32] Tobias J, Kassem E, Rubinstein U, Bialik A, Vutukuru SR, Navaro A, et al. Involvement of main diarrheagenic Escherichia coli, with emphasis on enteroaggregative E. coli, in severe non-epidemic pediatric diarrhea in a high-income country. BMC infectious diseases 2015;15:79. 10.1186/s12879-015-0804-4 25887696 PMC4339106

[33] Khalil IA, Troeger C, Blacker BF, Rao PC, Brown A, Atherly DE, et al. Morbidity and mortality due to shigella and enterotoxigenic Escherichia coli diarrhoea: the Global Burden of Disease Study 1990–2016. Lancet Infect Dis 2018;18:1229–1240. 10.1016/S1473-3099(18)30475-4 30266330 PMC6202441

[34] Duffy G, Burgess CM, Bolton DJ. A review of factors that affect transmission and survival of verocytotoxigenic Escherichia coli in the European farm to fork beef chain. Meat Sci 2014;97:375–383. 10.1016/j.meatsci.2014.01.009 24548772

